# Impaired Cellular Responses to Cytosolic DNA or Infection with *Listeria monocytogenes* and Vaccinia Virus in the Absence of the Murine LGP2 Protein

**DOI:** 10.1371/journal.pone.0018842

**Published:** 2011-04-14

**Authors:** Darja Pollpeter, Akihiko Komuro, Glen N. Barber, Curt M. Horvath

**Affiliations:** 1 Department of Molecular Biosciences, Northwestern University, Evanston, Illinois, United States of America; 2 Department of Medicine and Sylvester Comprehensive Cancer Center, University of Miami School of Medicine, Miami, Florida, United States of America; French National Centre for Scientific Research, France

## Abstract

Innate immune signaling is crucial for detection of and the initial response to microbial pathogens. Evidence is provided indicating that LGP2, a DEXH box domain protein related to the RNA recognition receptors RIG-I and MDA5, participates in the cellular response to cytosolic double-stranded DNA (dsDNA). Analysis of embryonic fibroblasts and macrophages from mice harboring targeted disruption in the LGP2 gene reveals that LGP2 can act as a positive regulator of type I IFN and anti-microbial gene expression in response to transfected dsDNA. Results indicate that infection of LGP2-deficient mice with an intracellular bacterial pathogen, *Listeria monocytogenes,* leads to reduced levels of type I IFN and IL12, and allows increased bacterial growth in infected animals, resulting in greater colonization of both spleen and liver. Responses to infection with vaccinia virus, a dsDNA virus, are also suppressed in cells lacking LGP2, reinforcing the ability of LGP2 to act as a positive regulator of antiviral signaling. *In vitro* mechanistic studies indicate that purified LGP2 protein does not bind DNA but instead mediates these responses indirectly. Data suggest that LGP2 may be acting downstream of the intracellular RNA polymerase III pathway to activate anti-microbial signaling. Together, these findings demonstrate a regulatory role for LGP2 in the response to cytosolic DNA, an intracellular bacterial pathogen, and a DNA virus, and provide a plausible mechanistic hypothesis as the basis for this activity.

## Introduction

Pathogen infection can be recognized by a variety of pattern recognition receptors (PRRs), including Toll-like receptors (TLRs), Nod-like receptors (NLRs), and RIG-I like receptors (RLRs), leading to the production of a wide variety of intrinsic defenses, including, but not limited to, the production of direct effectors of anti-microbial actions, interferons, cytokines, and chemokines [1]. Cell-autonomous responses are critical for the early detection of pathogen invasion and build the first barrier for microbial infections. For example, virus infection can lead to type I IFN induction in virtually all cell types and the antiviral activities of type I IFN can directly limit virus replication [2]. Infection also triggers paracrine antiviral signaling, as well as attraction and activation of immune cells to the site of infection. Large quantities of type I IFN are produced by plasmacytoid dendritic cells to further modulate appropriate adaptive immune responses and immune cell development and maturation [3]. These immune responses collaborate to eliminate the pathogen and produce specific and long lasting immunity [4].

Among the specific pathogen-associated molecular patterns (PAMPs) that can be detected by PRRs are pathogen-derived nucleic acids. The nucleic acid sensing transmembrane TLRs, TLR 3, 7, 8 and 9, localize to intracellular compartments and have well known ligand specificities, with TLR3 detecting dsRNA, TLR7 and TLR8 detecting ssRNA, and TLR9 responding to patterns found in microbial DNA and oligodeoxynucleotides encoding unmethylated CpG motifs [5]. In contrast, cytosolic RNA ligands are recognized by the RLR members retinoic acid-inducible gene I, RIG-I, and melanoma differentiation-associated gene 5, MDA5 [6], [7].

Upon engagement with non-self nucleic acids, RLRs activate serine kinase signaling cascades that converge on interferon (IFN) regulatory factor (IRF) and nuclear factor-κB (NF-κB) transcription factors, resulting in expression of IFNs, including type I IFN, anti-microbial effector genes, and inflammatory cytokines, though the relative composition and intensity of the response can vary depending on the properties and abundance of the PAMPs as well as the cell type and abundance of the intrinsic PRR signaling components [8].

In contrast to the rapidly growing understanding of cellular responses to pathogens with RNA genomes or pathogen-derived cytosolic RNA via the RLR pathways, the responses to cytosolic pathogens with DNA genomes remain enigmatic. Cytosolic delivery of double-stranded oligodeoxynucleotides lacking contiguous CpG sequences (IFN stimulatory DNA, ISD [9]), or right-handed helical B-form DNA (B-DNA, such as poly(dA-dT) [10]) can induce type I IFN expression independent of TLR pathways. Intracellular DNA-mediated signaling is dependent on TBK1 and IRF-3 in a variety of cell types including fibroblasts, dendritic cells and macrophages, suggesting the presence of unidentified cytosolic DNA receptor signaling systems [9], [10], [11]. An endoplasmic reticulum resident protein, stimulator of IFN gene (STING) has been identified as an important adaptor molecule for the sensing of cytosolic nucleic acids from both RNA and DNA viruses [12]. Murine cells deficient in STING have a defect in IFNβ production in response to cytosolic B-DNA as well as some DNA pathogens [13]. DNA-dependent activator of IRFs (DAI, also known as ZBP-1) has been characterized to recognize B-DNA directly and physically associate with TBK1 and IRF-3, resulting in activation of the IFNβ promoter [14]. Further analysis has revealed that B-DNA-induced oligomerization of DAI is crucial for signaling [15], but it may be required only for specific cell types and is notably absent in mouse embryonic fibroblasts (MEFs) [11], [15]. It is likely that additional proteins are involved in mediating and regulating the cellular responses to cytosolic dsDNA. In addition to direct sensing of pathogen dsDNA, it has been reported recently that dsDNA (poly(dA-dT)) can be transcribed by the endogenous cellular RNA polymerase III (polIII) to produce immunogenic RNA species in the cytosol of both human and mouse cells [16], [17]. This RNA is double-stranded and carries a 5′-triphosphate moiety, two features recognized by RLRs [18], [19].

Two of the RLR RNA detectors, RIG-I and MDA5, are characterized by a core DEXH box domain fused to tandem N-terminal caspase activation and recruitment domain (CARD) motifs that are essential for propagating downstream signal transduction. A third related RLR protein, LGP2, has high sequence similarity to MDA5 and RIG-I DEXH box domains but lacks the N-terminal CARD homology. Expression of LGP2 from a plasmid vector acts as a negative regulator of IFN production and antiviral signaling [6], [20], [21], [22], but analysis of mice deficient for LGP2 indicated disparate functions for LGP2 in response to different viruses [23]. Recent evidence suggests LGP2 acts as a positive regulator of IFN responses induced by diverse viruses and may act in concert with MDA5 and RIG-I [24]. In the present study, we explore a potential role for LGP2 in cellular responses to dsDNA and/or pathogens with DNA genomes. Our findings demonstrate that LGP2, though not essential, is required for generation of optimal cytokine responses to intracellular dsDNA, infection with the bacterium *Listeria monocytogenes* (LM) and the pox virus, modified vaccinia virus Ankara (MVA). Results from infection of mice with a targeted disruption in LGP2 indicate that LGP2-mediated responses contribute to limitation of LM growth *in vivo*. Results implicate an indirect mechanism for LGP2 action involving the RNA polymerase III pathway. This is the first report indicating that the DEXH box helicase LGP2 plays a role in the positive regulation of type I IFNs and other cytokines in response to intracellular B-DNA and both bacterial and viral DNA pathogens.

## Results

### LGP2 contributes to cytosolic dsDNA signaling

**Figure 1:**
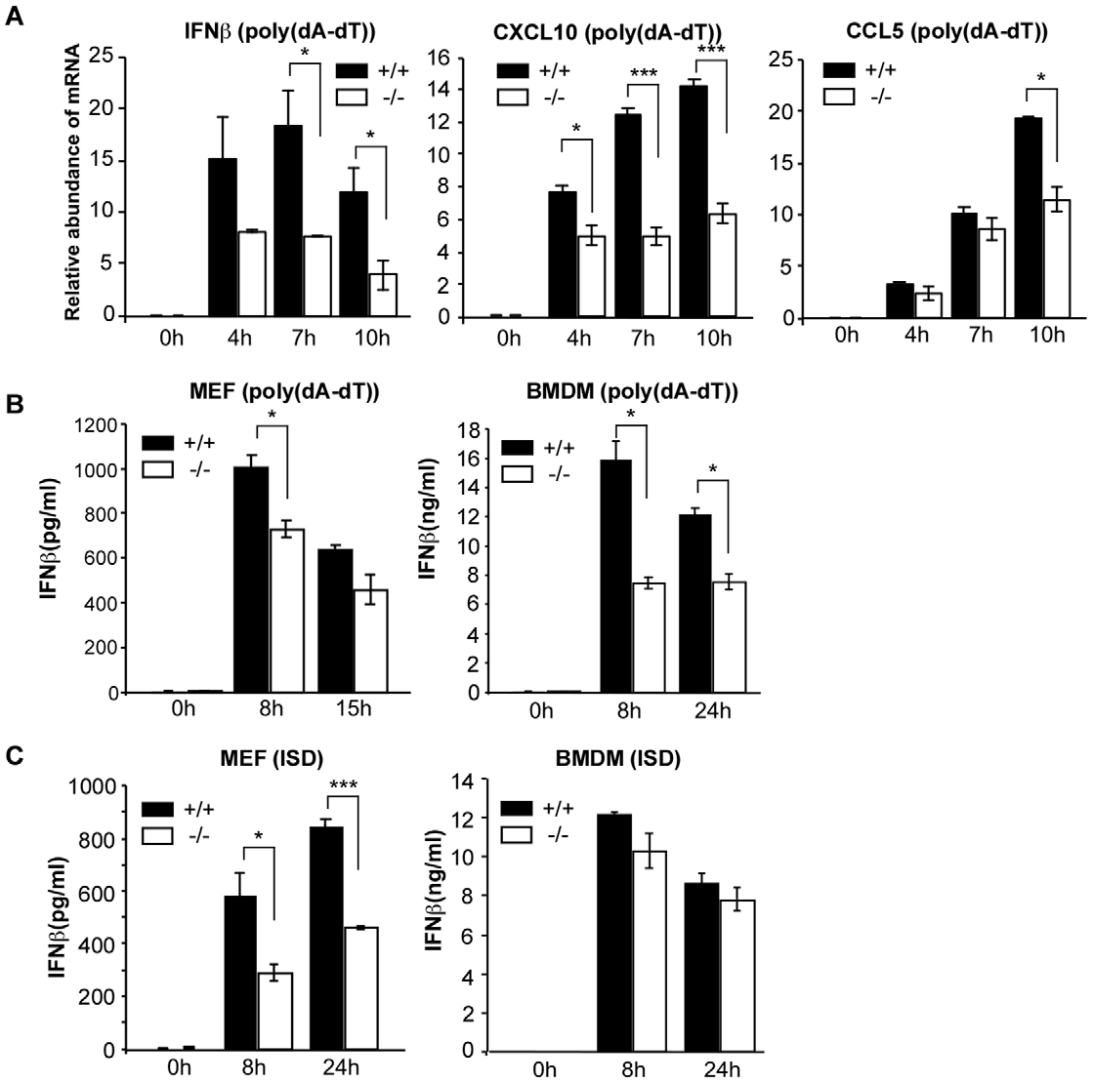
LGP2 deficiency decreases intracellular dsDNA signaling. (a) MEFs from wild-type mice (+/+) and LGP2-deficient mice (−/−) were transfected with 5 µg/ml of poly(dA-dT) for the indicated amount of time and total RNA was isolated from cells. The relative abundance of *IFNβ*, *CXCL10*, and *CCL5* mRNA was determined by quantitative RT-PCR. Error bars indicate standard deviation for duplicate PCR reactions. (b) MEFs or BMDM from wild-type mice (+/+) and LGP2-deficient mice (−/−) were transfected with 5 µg/ml poly(dA-dT) or (c) ISD. At indicated time points after transfection, media was collected and IFNβ levels were quantified by ELISA. Results are representative of at least 2 independent transfection experiments carried out in duplicate. Student’s t test was performed: *(p<0.05), **(p<0.01), and ***(p<0.005).

To determine if LGP2 contributes to cytosolic dsDNA signaling, embryonic fibroblasts from wild-type or LGP2-deficient mice [23] were transfected with poly(dA-dT) and the expression of RLR responsive genes was evaluated by real time RT-PCR. As indicators of anti-microbial signaling, the degree of accumulation of mRNAs for IFNβ, CXCL10, and CCL5 was evaluated. Both wild-type and LGP2-deficient MEFs responded to dsDNA transfection and accumulation of mRNAs for IFNβ, CXCL10, and CCL5 was observed, but the response was significantly reduced in the absence of LGP2 (Figure 1a). Measurement of secreted IFNβ confirmed the suppressed poly(dA-dT) response in MEFs as well as in bone marrow-derived macrophages (BMDM) from LGP2-deficient mice (Figure 1b). Upon transfection of interferon stimulatory DNA (ISD) no significant difference of IFNβ was found in BMDM in agreement with prior studies. However, IFNβ secretion from LGP2-deficient MEFs was reduced by approximately 50%. Together, these data suggest that LGP2 contributes to the positive regulation of dsDNA-mediated cellular innate immune responses.

### LGP2 deficiency decreases cellular responses to *Listeria monocytogenes*

**Figure 2:**
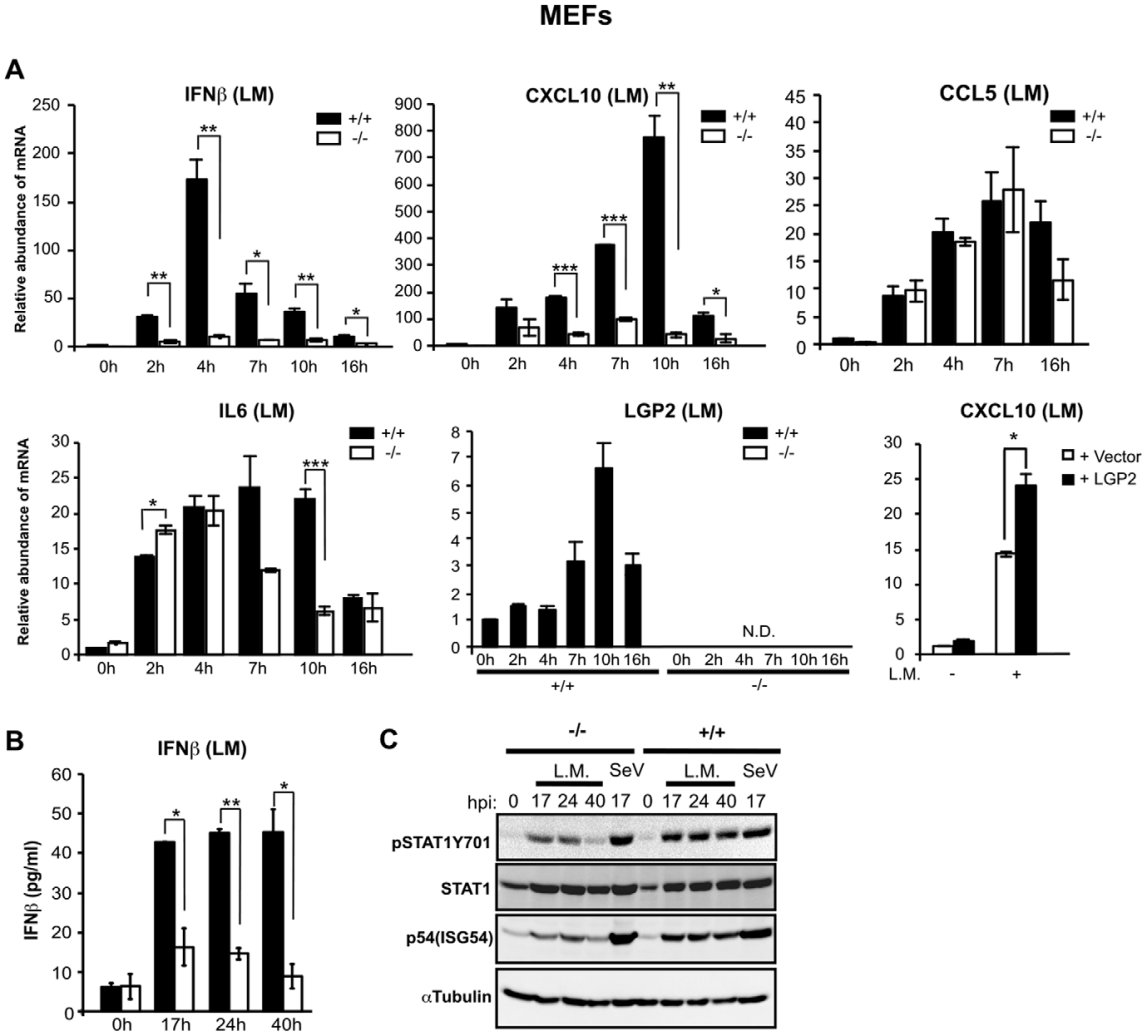
LGP2 positively regulates *Listeria monocytogenes* infection-mediated IFN and cytokine induction in MEFs. (a) MEFs from wild-type mice (+/+) and LGP2-deficient mice (−/−) were infected with LM for indicated amount of time and total RNA was isolated. Relative abundance of *IFNβ*, *CXCL10*, *CCL5*, *IL-6* and *LGP2* mRNA was determined by quantitative RT-PCR. MEFs from LGP2-deficient mice (−/−) were transfected with an expression plasmid for human LGP2, p3xFLAG-LGP2, or empty vector (p3xFLAG-CMV10) using Lipofectamine 2000. After 24 h cells were infected with LM for 4 h and RNA was isolated for quantitative RT-PCR to determine the relative abundance of *CXCL10* mRNA. (b) MEFs from wild-type mice (+/+) and LGP2-deficient mice (−/−) were infected with LM for the indicated amount of time and cell supernatant was analyzed for quantification of IFNβ by ELISA.(c) MEFs from wild-type mice (+/+) and LGP2-deficient mice (−/−) were mock infected, infected with LM or Sendai Virus (Cantell strain, m.o.i of 1 pfu/cell) for the indicated amount of time and cell lysates were analyzed by Western blot with indicated antibodies. Student’s t test was performed: *(p<0.05), **(p<0.01), and ***(p<0.005).

Genomic DNA is an important component of innate recognition and response to the intracellular bacterial pathogen *Listeria monocytogenes* (LM), and LM dsDNA has been characterized as a PAMP recognized in the cytosol of infected cells [9]. To examine the ability of LGP2 to mediate responses to this intracellular pathogen, wild-type and LGP2-deficient MEFs were infected with LM and cellular responses monitored. Real time RT-PCR was used to test the induction of RLR responsive IFNβ, CXCL10, and CCL5 mRNAs as well as IL6 and LGP2 itself (Figure 2a). All mRNAs accumulated in the infected cells. Induction of IFNβ and CXCL10 mRNAs was severely reduced in the LGP2-deficient cells, but expression of IL6 and CCL5 were largely independent of LGP2, although some reduction in their accumulation was also observed at later time points in the absence of LGP2. Importantly, the LGP2-deficient cells were complemented by expression of human LGP2, reversing the defect by restoring greater CXCL10 mRNA accumulation (Figure 2a). Consistent with the mRNA levels, IFNβ secretion into the medium was also defective in the absence of LGP2 (Figure 2b). Accordingly, immunoblotting demonstrated decreased IFN signal transduction in the absence of LGP2, resulting in decreased levels of STAT1 tyrosine phosphorylation and reduced expression of the ISG54 (p54) protein (Figure 2c). Together, these results demonstrate that LGP2 acts as a positive regulator of IFN production and downstream signaling induced by LM in MEFs.

### LGP2-deficient macrophages are defective for intracellular responses to *Listeria monocytogenes,* but not TLR-mediated signaling

Innate immune responses against LM have been well characterized in macrophages [9], [25], [26], [27], [28], and have been demonstrated to rely on cytosolic invasion catalyzed by the bacterial toxin listeriolysin O [29], [30] via a response pathway that is independent of TLRs and IPS-1/MAVS, an RLR adaptor molecule, but dependent on expression of the transcription factor IRF3 [9], [10], [31], [32].

**Figure 3:**
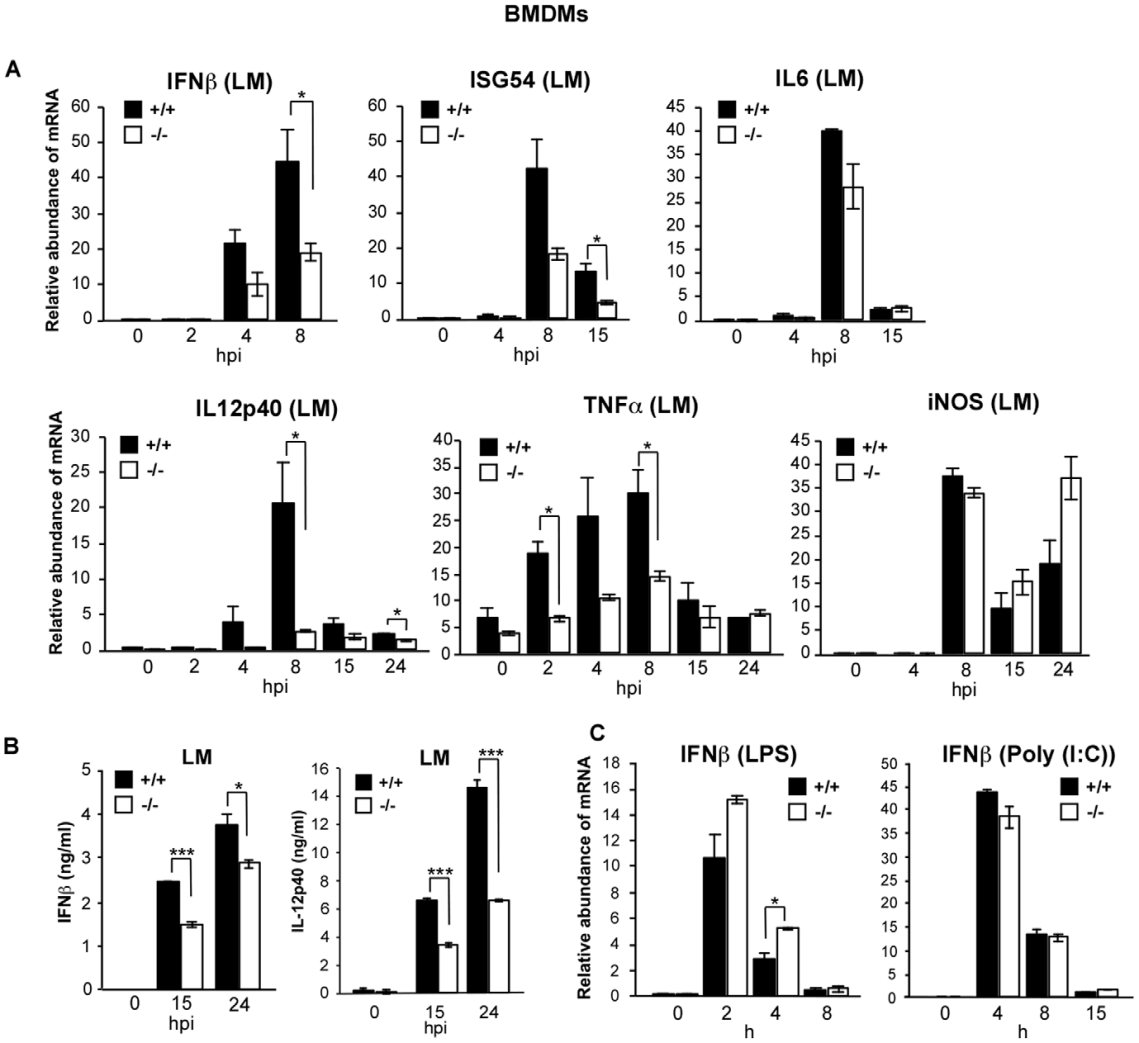
LGP2 deficiency interferes with *Listeria monocytogenes* infection-mediated immune responses in BMDMs. (a) BMDMs from wild-type mice (+/+) and LGP2-deficient mice (−/−) were infected with LM for indicated amount of time and total RNA was isolated. RNA was analyzed by quantitative RT-PCR to detect the relative abundance of *IFNβ, ISG54, IL6, IL12-p40, TNFα* and *iNOS* mRNA levels. (b) BMDMs from wild-type mice (+/+) and LGP2-deficient mice (−/−) were infected with LM for indicated amount of time and cell supernatant was analyzed for IFNβ and IL-12p40 by ELISA. (c) As controls, BMDMs were treated with TLR 4 ligand LPS (10 ng/ml) or TLR3 ligand Poly(I:C) (100 µg/ml) for indicated time points and the relative abundance of IFNβ mRNA was determined. Student's t test was performed: *(p<0.05), **(p<0.01). and ***(p<0.005).

To assess the role of LGP2 in macrophage type I IFN induction, bone marrow-derived macrophages (BMDMs) were prepared and infected with LM. Similar to our observations in MEFs, LM-induced IFNβ mRNA induction is reduced in LGP2-deficient BMDMs (Figure 3a), confirmed by the defective accumulation of ISG54. Similar assays for effects of LGP2 deficiency were executed for additional genes known to be important for limiting LM growth in the host including IL12p40, tumor necrosis factor alpha (TNFα), and inducible nitric oxide synthase (iNOS) [33], [34], [35]. Notably, induction of IL12p40 mRNA was severely reduced in LGP2-deficient macrophages and TNFα induction was also diminished by approximately 50%, while iNOS induction appeared largely unaffected (Figure 3a). The differential cytokine expression patterns were confirmed by measuring IFNβ and IL12 secreted into the culture medium in response to LM infection. Secretion of both cytokines in response to LM infection was impaired in the absence of LGP2 (Figure 3b).

To test the specificity of LGP2 for responses to cytosolic PAMPs, Toll-like receptor responses were also evaluated in the macrophages by using ligands specific for TLR3 and TLR4. Addition of either poly(I:C) or LPS to the culture medium resulted in IFNβ gene induction levels that were comparable between LGP2-deficient and wild-type cells (Figure 3c).

### LGP2 deficiency leads to reduced cytokine responses and enhanced colonization during *Listeria monocytogenes* infection *in vivo*

**Figure 4:**
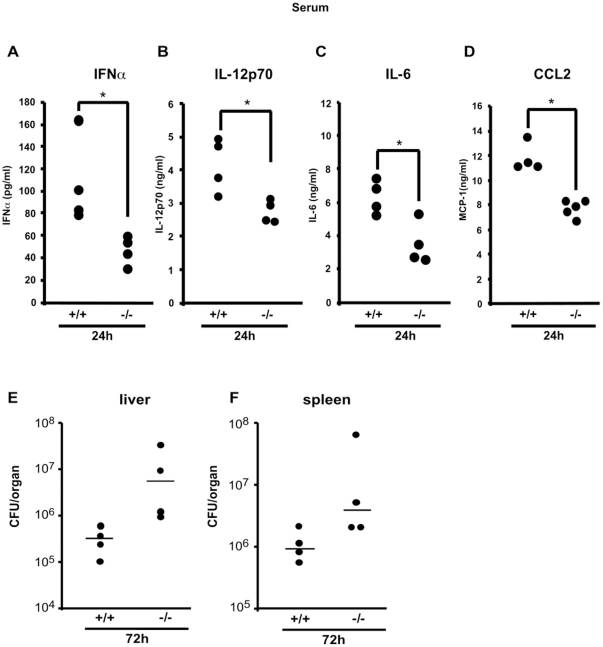
LGP2 increases *Listeria monocytogenes* infection-mediated cytokine and chemokine secretion and contributes to limitation of LM growth *in vivo*. Serum from wild-type (+/+) or LGP2-deficient (−/−) mice (n = 4–5 each) infected with LM were collected 24 h after infection. Serum IFNα (a) and IL-12p70 (b) levels were analyzed by ELISA and IL-6 (c), CCL2 (d) levels were analyzed by Milliplex assay (Millipore). Plotted dots indicate average cytokine concentration of duplicate readings from each mouse. Student's t test was performed: *(p<0.05), **(p<0.01), and ***(p<0.005). (e,f) Wild-type (+/+) and LGP2-deficient mice (−/−) (n = 4) were infected with 1.5×10^6^ wild-type LM and bacterial growth in the liver (e) and spleen (f) was measured 72 h after inoculation. The median of colony forming units (CFUs) from each group is indicated by bars. A representative experiment of two independent experiments is shown.

The extent to which LGP2 alters the response to LM infection and consequences on cytokine signaling was further explored *in vivo*. Both wild-type and LGP2-deficient mice were infected with 1x10^6^ CFU of LM via tail vein injection. After 24h, the level of IFNα was lower in the serum of infected LGP2-deficient mice compared to wild-type mice (Figure 4a). Similarly, IL12p70, IL-6 and CCL2 levels were all detected at a higher level in the serum of wild-type mice and were reduced in LGP2-deficient mice (Figure 4b-d). In all cases, the cytokine levels returned to near basal levels by 72 hours (not shown).

Together, these data indicate that the LGP2 protein participates in the regulation of innate responses including cytokine production in response to infection with LM *in vivo*. To address the potential ability of LGP2 and its downstream effects to contribute to the control of LM growth *in vivo*, livers and spleens of infected wild-type or LGP2-deficient mice were analyzed for bacterial colonization. At 72h post inoculation, an approximately 17-fold increase in median colony forming units per liver was detected in infected LGP2-deficient mice, and approximately 4-fold increase in median colony forming units per spleen was detected in the LGP2-deficient mice compared to wild-type mice (Figure 4e,f). These findings correlate well with a role for LGP2 as a regulator of responses to LM infection and pathogenesis.

### LGP2 deficiency decreases cellular responses to DNA Virus infection

**Figure 5:**
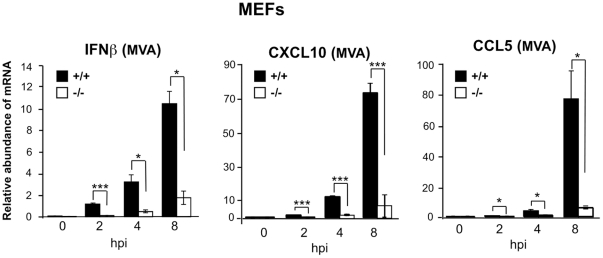
LGP2 positively regulates MVA-induced IFN and cytokine induction in MEFs. MEFs from wild-type mice (+/+) and LGP2-deficient mice (−/−) were infected with MVA at a multiplicity of infection of 10 for the indicated amount of time and total RNA was isolated. Relative abundance of *IFNβ*, *CXCL10* and *CCL5* mRNA was determined by quantitative RT-PCR. A representative experiment of three independent experiments is shown. Student's t test was performed: *(p<0.05), **(p<0.01), and ***(p<0.005).

In order to test the involvement of LGP2 in cellular innate immune responses to other pathogens with DNA genomes, we measured cytokine mRNA levels of wild-type and LGP2-deficient MEFs infected with a double-stranded DNA virus the poxvirus, modified vaccinia virus Ankara (MVA). Levels of IFNβ, CXCL10 and CCL5 are induced by infection at all time points but the response is impaired in LGP2-deficient cells compared to wild-type. This effect is especially dramatic in the levels of CXCL10 and CCL5 responses (Figure 5). These findings suggest a more general role for LGP2 in cellular innate immune responses to a variety of DNA pathogens.

### LGP2 does not interact directly with DNA

**Figure 6:**
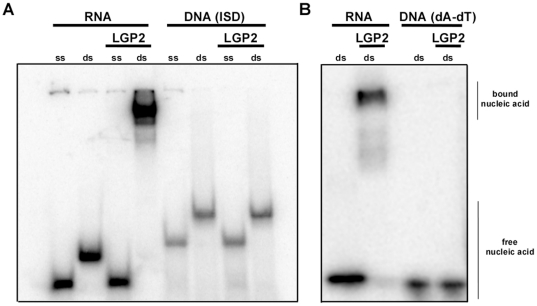
LGP2 selectively interacts with dsRNA but not dsDNA. Electrophoretic mobility shift assay of LGP2 with RNA or DNA. a) Single stranded (ss) or double-stranded (ds) RNA (27-mer) or DNA (ISD,45-mer) or b) dsRNA (27-mer) or ds(dA-dT) (28-mer) were incubated with or without LGP2 and analyzed by native PAGE and autoradiography.

**Figure 7:**
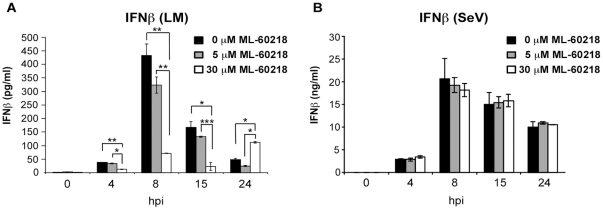
*Listeria monocytogenes-*induced IFN production in BMDM is dependent on RNA Polymerase III. BMDM from wild-type mice were treated with the indicated concentrations of RNA polymerase III inhibitor ML-60218 for 10 h and then infected with (a) LM (5 cfu/cell) or (b) Sendai Virus, Cantell Strain (5 pfu/cell) for indicated amounts of time and cell supernatants were analyzed for secreted IFNβ by ELISA. Student's t test was performed: *(p<0.05), **(p<0.01), and ***(p<0.005).

The implication of LGP2 in regulating signaling mediated by cytosolic DNA and pathogens with DNA genomes was further examined on a molecular mechanistic basis. To determine if LGP2 could directly participate in dsDNA recognition, an electrophoretic mobility shift assay was conducted with purified LGP2 protein. The protein was tested for the ability to form a stable complex with short ssRNA, dsRNA, and two dsDNAs, poly(dA-dT) and ISD. While LGP2 bound well to the dsRNA probe, no stable complex was formed with ssRNA or either DNA probe (Figure 6). These data suggest that LGP2′s regulatory role in cellular responses to dsDNA is not due to direct DNA recognition. One indirect pathway that could reconcile these seemingly dichotomous observations is the recently-described RNA polymerase III-mediated signaling system, where transfected poly(dA-dT) is transcribed by RNA polymerase III and the transcription products are recognized by the intracellular RLR protein system [16], [17]. This polIII activity was implicated in the detection of DNA pathogens, including *Legionella pneumophila* and HSV-1, using pharmacologic inhibition of cellular RNA polymerase III. To determine if the polIII-mediated process could potentially play a role in LGP2 function in this context, we investigated whether LM-induced cellular responses are also affected by inhibition of RNA polymerase III. BMDM were treated with the RNA polymerase III inhibitor, ML-60218, then infected with LM or with the RNA virus, Sendai virus, as a control. ML-60218 did not affect IFNβ levels secreted by macrophages after Sendai virus infection, but dramatically reduced IFNβ levels resulting from LM infection (Figure 7). These findings suggest that LM can trigger intracellular signaling at least in part via the RNA polymerase III pathway to create RLR ligands. Since LGP2 is known to positively regulate RLR signaling [24], we hypothesize that the observed effects of LGP2 deficiency in poly(dA-dT) signaling and DNA pathogen responses could plausibly result from a failure to optimally process the RNA products downstream of RNA polymerase III.

## Discussion

The experiments described in this study indicate a regulatory role for the DEXH box protein, LGP2, in the innate cellular immune response to intracellular dsDNA and infection with the microbial pathogens *Listeria monocytogenes* and modified vaccinia virus Ankara. We have observed that cytosolic DNA-induced type I IFN production is attenuated in the absence of LGP2 in several cell types, and demonstrate that responses to LM and dsDNA virus infection are also defective in the absence of LGP2. These data suggest that LGP2 is not essential for cytosolic DNA-mediated responses but is required for maximal cytokine induction.

The role of LGP2, which has high sequence similarity with RIG-I and MDA5, selectively binds dsRNA, but lacks CARD effector domains for IPS-1 signaling, has been under intense investigation. Several initial studies with LGP2 expression from exogenous expression plasmids characterized LGP2 as a negative regulator of dsRNA signaling [20], [21], [22], [36]. Similarly, we observed an inhibition of dsDNA-induced cytokine production by overexpression of LGP2 (unpublished observation). In contrast, the first study on LGP2-deficient mice and cells infected with RNA viruses or challenged with synthetic dsRNA poly(I:C), suggested a disparate function with LGP2 supporting signaling through MDA5 ligands but inhibiting RIG-I-mediated signaling [23]. A second independent study on LGP2-deficient mice, however, reported that LGP2 deficiency decreased cytokine responses to infection with several RNA viruses [24]. In this report, it was surmised that LGP2 may function upstream of RIG-I and MDA5 and its adaptor, IPS-1, as a positive regulator of antiviral responses. Due to these partially contrasting results, the exact role of LGP2 required further characterization. Our data expand the role of LGP2 to DNA pathogen detection within a cell and further corroborate the idea of a positive regulatory role for LGP2 in cytokine response to cytosolic stimuli.

The observation that DNA signaling responses were not entirely eliminated in the absence of LGP2 is consistent with a regulatory or supporting role for LGP2 in DNA responses that are at least partially redundant. In this respect, our findings are consistent with previous studies implicating the MDA5/IPS-1 pathway in addition to TLR-mediated sensing for optimal cytokine responses to MVA infection [37], [38]. Interestingly siRNA-mediated knockdown of MDA5 and IPS-1 but not RIG-I impaired interferon responses to MVA infection [37]. Conversely, similar knockdown experiments showed that interferon responses to poly(dAdT) are primarily dependent on RIG-I and not MDA5 [16], [17], [39]. Our results therefore support the idea that LGP2 positively contributes to both RIG-I and MDA5-mediated signaling.

Our observation that LM-induced type I IFN production is mediated in part by RNA polymerase III adds to the growing list of diverse machinery used by the host to respond to LM [40], [41], [42], [43]. We propose a model in which segments of the AT-rich LM genome could be transcribed by RNA polymerase III into RNAs that can serve as ligands for RLR signaling. The potency of this signal is determined in part by the availability of cellular LGP2. As such, these studies add to the growing understanding of LGP2 as a complex regulator of intracellular pathogen-induced signal transduction, and suggest that it may be a node or branch point for coordinating crosstalk among seemingly diverse innate responses that are activated in infected cells. Further research will be required to fully investigate this hypothesis, including identification of the putative immunogenic RNA transcripts derived from the DNA pathogens.

In sum, this study has identified LGP2 as a component of innate cellular responses mediated by cytosolic dsDNA and DNA pathogens, and demonstrated that LGP2 is playing an unanticipated role in the positive regulation of downstream responses *in vitro* and *in vivo*.

## Materials and Methods

### Mice, Cells, *Listeria monocytogenes*, Viruses and Reagents

LGP2-deficient (-/-) mice were as described [23]. All animal experimentation was conducted following the NIH guidelines for housing and care of laboratory animals and performed in accordance with institutional regulations after review and approval by the Institutional Animal Care and Use Committee at University of Miami, School of Medicine (IACUC protocol 09-116). Bone marrow cells were prepared from 8 to 12-week-old LGP2 wild-type (+/+) and LGP2-deficient (−/−) mice. Mice used in *in vivo* experiments were 7 to 9-week old in age and were tested in sex-matched pairs. *Listeria monocytogenes* wild-type strain 43251 was purchased from ATCC. Sendai virus (SeV) Cantell strain was provided by Tom Moran (Mount Sinai, NY). Modified vaccinia virus Ankara (MVA) was provided by Joao Marques (Northwestern University). LPS from *E. coli* was purchased from Sigma-Aldrich. Poly(dA-dT)•poly(dT-dA) (poly(dA-dT)) and poly(I:C) were purchased from GE healthcare. ISD and dAdT oligos were synthesized by MWG-biotech and annealed as described [9]. The mouse ISG54 antibody was a kind gift from Ganes Sen (Cleveland Clinic). STAT1 and phosphorylated STAT1 antibodies were purchased from Santa Cruz Biotechnology and Cell Signaling, respectively. α-tubulin antibody was purchased from Calbiochem. Gentamycin sulfate and Lipofectamine 2000 were purchased from Invitrogen. The RNA polymerase III inhibitor ML-60218 was purchased from Calbiochem and used as described [16].

### Preparation of macrophages

To prepare bone marrow macrophages, cells were cultured with DMEM supplemented with 30% L cell conditioned media, 20% FBS, and primosin (Invivogen). After 7–9 days, cells were collected and used as bone marrow-derived macrophages (BMDMs).

### Electrophoretic mobility shift assay

Oligoribonucleotides (27 ntd), DNA oligos (45 ntd, ISD sequence) or dAdT oligonucleotide (28 ntd) were radiolabeled by polynucleotide kinase (NEB) and γ-^32^P ATP (Perkin Elmer). The labeled single stranded oligonucleotides were annealed to their complement and incubated for 30 min at room temperature with immuno-purified LGP2 derived from recombinant baculovirus as described previously [44]. Reactions were separated on 10% native polyacrylamide gels, dried and detected by phosphorimage analysis.

### *Listeria monocytogenes* growth and infections

*Listeria monocytogenes* strain 43251 were grown to mid-log in brain heart infusion media (Difco, BD) in a shaking incubator at 37°C. Bacteria were then washed and resuspended in PBS. BMDMs were infected with LM strain 43251 at 2–5 bacteria per cell. MEFS were infected at 80 bacteria per cell. 1 h post infection gentamycin (50 µg/ml) was added for 1 h to kill extracellular bacteria before the medium was changed to regular media containing 25 µg/ml gentamicin and primosin. For bacteria titers in liver and spleen and for serum cytokine analysis mice were injected intravenously with 1×10^6^ LM cells.

### Virus infections

MEFs were seeded at 2×10^5^ cells/well in a 12 well format and infected with MVA at a multiplicity of infection of 10 Pfu/cell in serum free media for 2 h, before media change to DMEM supplemented with 2% CCS. Cells were harvested with Trizol (Invitrogen) for RNA extraction.

### Western Blot Analysis

MEFs were lysed in a WCE buffer containing 50 mM Tris-HCl (pH 8.0), 280 mM NaCl, 0.5% NP-40, 0.2 mM EGTA, 2 mM EDTA, 10% glycerol, 1 mM DTT, 1 mM NaF, 1 mM Na_3_VO_4_ and protease inhibitor cocktail (Calbiochem). The cell lysates were resolved by SDS-PAGE and transferred onto a nitrocellulose membrane. The membrane was blotted with the specific antibody to the indicated protein, and visualized with an enhanced chemiluminescence system (NEN Life Science Product).

### Measurement of cytokine production

MEFs (1×10^5^) and BMDMs (2×10^5^) were infected as indicated. For analysis of IFNα IFNβ IL-12p40, and IL-12p70 production, supernatants from cell culture or serum from mice were collected at indicated time points after infection and used for a sandwich ELISA or Milliplex cytokine measurement. ELISA kits for mouse IFNα and IFNβ were purchased from PBL Biomedical Laboratories and IL-12p40 and IL-12p70 were from R&D Systems. Mouse cytokine assay Milliplex MAP kit for measurement of IL-6, MCP-1 (CCL2), and IL-12 was purchased from Millipore.

### Quantitative Real-Time Reverse Transcriptase (RT) PCR

For quantitative PCR, cells were harvested with TRIzol (Invitrogen) or RNeasy kit (QIAGEN). RNA was reverse transcribed with Superscript III (Invitrogen), and cDNAs were used for PCR with QuantiTect SYBR Green reagents (Invitrogen) on a Stratagene MX3000. The abundance of each cytokine mRNA was normalized to GAPDH mRNA levels with ΔΔCT method. Primer sequences are as follows: mouse *lgp2*-forward primer: 5′-GAGACCTGGAGGAACCATCA-3′, mouse *lgp2*-reverse primer: 5′-CCCTCGAGGTGTTTCCAGTA-3′; mouse *ccl5-*forward primer: 5′-GCCCACGTCAAGGAGTATTT-3′, mouse *ccl5-*reverse primer: 5′-TCGAGTGACAAACACGACTG-3′; mouse *cxcl10*-forward primer: 5′-GGATGGCTGTCCTAGCTCTG-3′, mouse *cxcl10*-reverse primer: 5′-TGAGCTAGGGAGGACAAGGA-3′; mouse *IFNβ*-forward primer: 5′-GCAGCTGAATGGAAAGATCA-3′, mouse *IFNβ*-reverse primer: 5′-TGGCAAAGGCAGTGTAACTC-3′; mouse *Il-6*-forward primer: 5′-CCGGAGAGGAGACTTCACAG-3′, mouse *Il-6-*reverse primer: 5′-TCCACGATTTCCCAGAGAAC-3′; mouse *gapdh*-forward primer: 5′-CAAGGAGTAAGAAACCCTGGACC-3′, mouse *gapdh*-reverse primer: 5′-CGAGTTGGATAGGGCCTCT-3′; mouse *IFNα2*-forward primer: 5′-AGCAGATCCAGAAGGCTCAA-3′, mouse *IFNα2*-reverse primer: 5′-GGAGGGTTGTATTCCAAGCA-3′; mouse *ISG54*-forward primer: 5′-GGAAAAAGAAAGCCCTCACC-3′, mouse *ISG54*-reverse primer: 5′-GTTCCCCAAACTCCTGACAA-3′; mouse *Il12p40*-forward primer: 5′-AGCAGTAGCAGTTCCCCTGA-3′, mouse *Il12p40*-reverse primer: 5′- AGTCCCTTTGGTCCAGTGTG-3′; mouse *inos*-forward primer: 5′-GACGAGACGGATAGGCAGAG-3′, mouse *inos*-reverse primer: 5′-GTGGGGTTGTTGCTGAACTT-3′; mouse *tnfα*-forward primer: 5′-ACGGCATGGATCTCAAAGAC-3′, mouse *tnfα*-reverse primer: 5′-GTGGGTGAGGAGCACGTAGT-3′.

### Statistical analysis

A two tailed unpaired Student’s t-test was performed and p-values are indicated with *(p<0.05), **(p<0.01), and ***(p<0.005).
